# Cross-Cultural Translation into Brazilian Portuguese and Validation of the Oral Anticoagulation Knowledge Tool (AKT-Br)

**DOI:** 10.21470/1678-9741-2020-0731

**Published:** 2022

**Authors:** Felipe F. Mainka, Vinicius L. Ferreira, Antonio M. Mendes, Gustavo L. Marques, Cassyano J. Correr, Fernanda S. Tonin, Roberto Pontarolo

**Affiliations:** 1 Pharmaceutical Sciences Postgraduate Programme, Universidade Federal do Paraná, Curitiba, Paraná, Brazil.; 2 Hospital de Clínicas, Universidade Federal do Paraná, Curitiba, Paraná, Brazil.; 3 Department of Pharmacy, Universidade Federal do Paraná, Curitiba, Paraná, Brazil.; 4 Research Institute for Medicines (iMed.ULisboa), Universidade de Lisboa, Lisbon, Portugal.

**Keywords:** Anticoagulants, Warfarin, Surveys and Questionnaires, Medication Adherence, Translating, Reproducibility of Results, Decision Support Techniques

## Abstract

**Introduction:**

Oral anticoagulants are the treatment of choice for diverse types of coagulation disorders. Warfarin is widely used by the Brazilian population, possibly due to its lower cost than other oral anticoagulants. However, it has a high risk of serious adverse effects if used incorrectly. The Anticoagulation Knowledge Tool (AKT) can assess a patient’s knowledge about her/his oral anticoagulant therapy and can assist health professionals in identifying patients with difficulties in adherence. This study aimed to translate, culturally adapt, and validate the AKT into Brazilian Portuguese.

**Methods:**

After a standard forward-backward procedure to translate the AKT into Brazilian Portuguese (AKT-Br), a version of the instrument was applied in three groups (patients, pharmacists, and the general population). The reliability of the AKT-Br was tested using an internal consistency measure and test-retest. The validity of the instrument was confirmed with data from the contrasted groups. All statistical analyses were performed with RStudio.

**Results:**

The median scores obtained with the AKT-Br were 29.0, 17.0, and 7.5 for pharmacists, patients, and the general population, respectively (maximum score of 35 points). There was moderate internal consistency for the instrument and test-retest reliability was satisfactory. Analysis of variance for validity of the groups revealed a significant relationship between the total score and the evaluated groups.

**Conclusion:**

The ATK-Br is a reliable and valid tool to assess knowledge about oral anticoagulants. AKT-Br can be used in clinical practice as an auxiliary tool to improve patient care through personalised educational interventions.

**Table:** 

Abbreviations, acronyms & symbols			
AKT	= Anticoagulation Knowledge Tool	IQR	= Interquartile range
AKT-Br	= Brazilian version of Anticoagulation Knowledge Tool	NA	= Not applicable
ANOVA	= Analysis of variance	OAC	= Oral anticoagulant therapies
CVI	= Content validity index	RNI	= Razão normalizada internacional
DOAC	= Direct oral anticoagulants	S-CVI	= Scale content validity index
I-CVI	= Item content validity index	VKA	= Vitamin K antagonists
INR	= International normalized ratio		

## INTRODUCTION

Atrial fibrillation and deep vein thrombosis are associated with substantial morbidity and mortality worldwide, resulting in medical and economic burdens^[1,2]^. Patients with atrial fibrillation have a fivefold increased risk of stroke and related death compared with patients without this condition^[3,4]^.

Oral anticoagulant therapies (OAC), which are broadly classified into vitamin K antagonists (VKA) and direct oral anticoagulants (DOAC), are highly effective for the management of thromboembolic disorders; their use reduces the risk of stroke and systemic embolism by around two thirds^[5,6]^. However, these therapies are considered high-risk medications. Although VKA have been used for more than 50 years, they require intensive coagulation monitoring, are characterised by wide variation in dose-response relationships, and have been associated with multiple drug-food and drug-drug interactions^[7,8]^. DOAC were recently introduced into clinical practice with the aim to overcome some of the limitations of VKA; however, some DOAC present narrow therapeutic windows, a factor that contributes to reduce adherence rates^[8]^.

Patients with great knowledge of their medication and clinical condition can participate in self-management, are more likely to adhere to treatment, and have a positive control of their coagulation compared with those with inadequate knowledge, in whom it is common to observe difficult coagulation control, increased risk of bleeding, and more readmissions^[9-13]^. In this context, a number of tools have been developed to assess a patient’s knowledge on OAC^[14,15]^, including the Anticoagulation Knowledge Tool (AKT) that covers patients who are prescribed DOAC or VKA.

The AKT measures a patient’s knowledge of her/his treatment. According to the results obtained with this tool, health professionals can select other interventions for the needs of each patient, such as providing guidance on diet, possible drug interactions, and the importance of keeping the international normalized ratio (INR) in an ideal range. However, evidence regarding the AKT psychometric proprieties, validity, and reliability are unavailable in non-English speaking settings, such as for the Brazilian Portuguese population. Translation and cross-cultural validation methods allow valid translations of psychometric tools from one language to another^[16,17]^. Thus, we aimed to provide further evidence on the validity of the AKT and to develop the Brazilian version of this tool (AKT-Br).

## METHODS

### AKT Description

The AKT is an instrument with evidence of validity and reliability of construct developed by Obamiro et al.^[14]^. It measures a patient’s knowledge of her/his anticoagulant therapy through self-administered questions and calculated scores. The AKT has 28 items (open-ended and multiple-choice questions) divided into two sections (A and B) with a maximum score of 35 points and 25 points, respectively, for VKA users and DOAC users. This difference between scores is because section B of the tool is exclusive dedicated for VKA users. Section B has eight items and a maximum score of 10 points. Section A (applied to all OAC users - both VKA and DOAC) presents 20 items and has a maximum score of 25 points. This section covers general questions about anticoagulants. To each given answer, a zero (incorrect answer) or a one (correct answer) is attributed by the clinicians to assess a patient’s knowledge^[14]^.

### Cultural and Linguistic Validation

We performed a culturally acceptable translation of AKT into Brazilian Portuguese. This step followed the International Society for Pharmacoeconomics and Outcomes Research (or ISPOR) recommendations^[17]^; it involved researchers to translate, back translate, apply, and validate the instrument.

A direct translation from English to Portuguese was performed by two independent Portuguese mother language translators (V.F, K.S), both with previous knowledge about anticoagulation. The consensus of this stage was reached through a discussion panel involving the two translators and the key-country consultant (R.P) and the project manager (F.M). The back-translation process was performed independently by two other translators (A.F, F.T), both without previous knowledge of the AKT, leading to a literal translation of the document. The consensus of this second stage occurred through a discussion panel involving R.P and F.M that compared the back translation with the original tool to finally obtain the AKT-Br version (see Supplementary Material).

Quantitative validity of the AKT-Br was achieved through the content validity index (CVI) and followed the minimum recommendations described by Lawshe^[18]^ and Lynn^[19]^. This phase involved a panel, with five anticoagulation experts, to obtain a level of agreement on the tool items. The clarity of the text, relevance, and quality of the back translation of each item were discussed. Items with a CVI < 1 were reassessed, deleted, or replaced. The clarity and relevance of the items were assessed using a four-point Likert scale (1 = not clear/not relevant; 4 = highly clear/highly relevant) and the back translation correspondence regarding the original instrument (1 = does not match; 4 = totally matches)^[19]^. The CVI was calculated using two approaches (item level [I-CVI] and scale level [S-CVI]), considering the average of the scores of the I-CVIs and error scale^[20]^. The I-CVI is the index that expresses the proportion of agreement among the evaluators for a given item and, according to Lynn^[19]^, in a panel of “five or fewer specialists, everyone must agree with the validity of the content for its classification to be considered a reasonable representation of the universe of possible classifications”. Any result < 100% agreement (< 1) deserves due attention and possible reassessment until reaching unanimous agreement among the evaluators. The S-CVI is equivalent to the average percentage of agreement between the items and, according to Waltz et al.^[21]^, the recommended standard acceptability criterion is 0.90.

### Assessment of Psychometric Proprieties

A cross-sectional study with convenience sampling was performed, following the protocol conducted in the original AKT^[14]^. The samples were obtained in a non-probabilistic manner for convenience sampling of three groups with different levels of knowledge about OAC: pharmacists (the pharmacist group), users of oral anticoagulants (the patient group), and the general population (the population group). Pharmacists were eligible to participate if they had at least one year of clinical experience or worked in community pharmacies. Patients were eligible if there were over 18 years of age and used any OAC. Eligible individuals from the general population were over 18 years old who were not receiving treatment with OAC or did not have relatives or close friends undergoing treatment with these therapies. Participants were informed of the anonymity and confidentiality of their responses. To participate in the study, participants needed to provide written informed consent. The exclusion criteria for patients were as follows: incomplete questionnaire completion and OAC treatment duration < 3 months. For illiterate patients or those with reading difficulties, the questions and answers options were read by the interviewers exactly as written, minimising possible bias, while for the others the questionnaires were self-administered^[22]^.

The questionnaires were applied from September 2019 to January 2020. Pharmacists were interviewed using an electronic form, while the general population was interviewed under the supervision of an interviewer; both groups were asked to respond based on their knowledge on anticoagulation. For the patient group, the questionnaires were applied in a tertiary hospital in the South Region of Brazil (Curitiba, Paraná) using the printed version of the AKT-Br and under the supervision of an interviewer.

### Statistical Analysis

The original 28-item structure of the AKT was maintained for all analyses^[14]^. Descriptive statistical analyses were performed to describe the characteristics of the sample. The variables age, treatment, gender, and educational level were considered non-normally distributed. The results are presented as relative and absolute frequencies for categorical variables and as the median and the interquartile range (IQR) (presented in square brackets) for continuous variables. Contrasted group analysis was conducted to assess differences in the mean scores between the three groups. One-way analysis of variance (ANOVA) with Tukey’s post hoc analysis was used to explore statistical differences^[23]^.

Statistical tests that assess internal consistency and test-retest reliability are commonly used in self-administered instruments to ensure the reliability of the instrument^[24]^. The evaluation of internal consistency was performed through Cronbach’s alpha coefficient that uses a scale of 0 to 1, where values close to 0.7 are considered acceptable and values > 0.9 are redundant^[25,26]^. In addition, for instruments with more than 15 items it is recommended to apply the interitem correlation^[27]^. The test-test reliability was obtained by reapplying the test to the same group, considering an appropriate time interval (14 days). Reliability coefficients between 0.7 and 0.8 (on a scale of 0 to 1) are considered acceptable^[24]^. The level of significance of each test was set at 0.05 (two-tailed). All statistical analyses were conducted using RStudio (version 1.3.1073).

### Ethics

The research was conducted within the standards required by the Declaration of Helsinki and approved by the Ethics Committee of the Hospital de Clínicas of the Universidade Federal do Paraná (Curitiba, Paraná, Brazil) under registration number CAAE: 16858719.1.0000.0096.

## RESULTS

### Cultural and Linguistic Validation

The final version of the AKT-Br (see Supplementary Material) was obtained through the consensus discussion of five anticoagulation specialists. Two hundred people were invited to participate in the study (75 pharmacists, 75 patients, and 50 people from the general population), of whom 148 met all eligibility criteria and were included for statistical analyses (55 pharmacists, 57 patients, and 36 people from the general population). Of this sample, 96 were women (64.9%), with a median age of 36 [IQR 28-53] years. About 67% of patients had used OAC for > 2 years (see Table 1).

**Table 1:** Demographic characteristics of the sample.

Group	N (total)	Female, N (%)	Age [IQR] (years)	Educational level, N (%)	Treatment duration, N (%)
Pharmacist	55	44 (80)	28 [26-33]	Bachelor 19 (34.5)	NA
				Postgraduate 36 (65.5)	
Patient	57	32 (56.1)	58 [46-68]	No formal education 8 (14)	< 3 months 2 (3.5)
				High school (36.8)	3-12 months 6 (10.5)
				College 14 (24.6)	12-24 months 9 (15.8)
				Technical education 4 (7)	> 24 months 40 (70.2)
				Bachelor 9 (15.8)	NA
				Postgraduate 1 (1.7)	NA
Population	36	20 (55.6)	29.5 [27-42.75]	College 5 (13.9)	NA
				Bachelor 16 (44.4)	
				Postgraduate 15 (41.7)	

IQR=interquartile range; NA=not applicable

### Quantitative and Qualitative Content Validity

The unilateral ANOVA did not reveal a correlation between the total score and age (F=4.8365; *P*<0.001), but there were correlations for gender, education, and group (F=10.0121, 11.0706, 172.0956, respectively; *P*<0.001), where women, individuals with higher education, and pharmacists presented the highest scores.

The evaluated items of the tool presented I-CVIs ranging from 0.7 to 1 and an S-CVI of 0.92 (see Table 2). Cronbach’s alpha for the pharmacist and patient groups were 0.71 and 0.65, respectively. The test-retest reliability resulted in r=0.99 (*P*<0.001). The textual analysis showed that the AKT-Br presents clear and relevant items, without the need for further modifications.

**Table 2:** Content validity index.

Panelists (N = 5)				
ITEM	I-CVI - clarity	I-CVI - relevance	I-CVI - back translation	S-CVI
**SECTION A**				
**Item 1** What is the name of your anticoagulant medicine? (Qual é o nome do seu medicamento anticoagulante?)	1	0.85	1	0.92
**Item 2** Why has your doctor prescribed you this medicine? (Por que seu médico prescreveu esse medicamento para você?)	0.95	1	1	
**Item 3** How does this medicine work in your body? (Você sabe como esse medicamento age em seu corpo?)	0.95	0.95	1	
**Item 4** How many times a day do you need to take this medicine? (Quantas vezes ao dia você precisa tomar esse medicamento?)	1	1	1	
**Item 5** For how long do you need to take this medicine (for example, 3 months, 6 months, life-long)? (Por quanto tempo você precisa tomar esse medicamento (por exemplo, 3 meses, 6 meses, por toda a vida?)	1	1	1	
**Item 6** Why is it important to take this medicine exactly as your doctor has told you? (Por que é importante tomar esse medicamento exatamente como o seu médico lhe explicou?)	0.95	0.9	0.9	
**Item 7** Is it important to take this medicine at the same time each day? (É importante tomar esse medicamento no mesmo horário todos os dias?)	1	1	1	
**Item 8** Is it okay to double the next dose of this medicine if you miss a dose? (Você acha que existirá algum problema se você dobrar a dose do anticoagulante caso tenha esquecido de tomar a dose anterior?)	1	0.9	1	
**Item 9** Is it possible that skipping one dose of this medicine could worsen your condition? (Você acha que esquecer uma dose do anticoagulante pode piorar a sua doença?)	0.85	0.85	1	
**Item 10** Is it appropriate to stop taking this medicine once you feel better? (Você acha apropriado parar de tomar o anticoagulante quando você se sente melhor?)	0.95	0.9	1	
**Item 11** Is it safe to take anti-inflammatory medicines like ibuprofen (Nurofen® or Advil®) while you are taking this medicine? (Você acha seguro tomar medicamentos anti-inflamatórios, como ibuprofeno (Alivium®, Ibupril® ou Advil®), enquanto você está tomando esse anticoagulante?)	0.95	0.95	1	
**Item 12** Is it safe to take vitamin supplements and herbal medicines with this medicine without consulting your doctor? (Você acha seguro tomar suplementos vitamínicos ou ervas medicinais com esse medicamento sem consultar seu médico?)	0.9	0.85	0.95	
**Item 13** Is there any benefit in taking more of this medicine than your doctor has told you to take? (Você acha que existe algum benefício em tomar doses acima da recomendada pelo seu médico?)	0.95	0.9	0.9	
**Item 14** Will drinking too much alcohol increase the risk of side effects with this medicine? (Você acha que tomar bebidas alcoólicas em grandes quantidades com esse medicamento aumenta o risco de efeitos colaterais?)	0.9	0.9	0.9	
**Item 15** Would you inform a surgeon, dentist or other health professional that you are taking this medicine before undergoing surgery or a procedure? (Você informaria um cirurgião, dentista ou outro profissional de saúde de que está tomando esse medicamento antes de realizar uma cirurgia ou um procedimento?)	1	1	1	
**Item 16** Is it important that all the health care practitioners you see know that you are taking this medicine? (É importante que todos os profissionais de saúde pelos quais você é acompanhado saibam que você faz uso de anticoagulante?)	0.95	1	1	
**Item 17** What is the most important side effect of this medicine? (Qual é o efeito colateral mais importante que seu anticoagulante pode causar?)	0.85	0.8	0.9	
**Item 18** THREE signs of side effects that you should watch out for while taking this medicine are: (Cite TRÊS sinais de efeitos colaterais para os quais você deve estar atento quando usa anticoagulante:)	0.75	0.75	0.9	
**Item 19** THREE things you can do to reduce your risk of side effects are: (Cite TRÊS coisas que você pode fazer para reduzir os riscos de efeitos colaterais dos anticoagulantes:)	0.9	0.7	0.95	
**Item 20** What is the best step to take if you accidentally take too much of this medicine? (Se acidentalmente você tomasse uma dose de anticoagulante muito acima da prescrita, o que você faria?)	0.75	0.8	0.9	
**SECTION B**				
**Item 1** What is your target INR range? (Qual é o valor de RNI ideal para você?)	0.75	0.8	0.9	
**Item 2** What was your last INR reading? (Qual foi seu último resultado de RNI?)	0.9	0.7	0.85	
**Item 3** Are regular INR tests necessary to know how well this medicine is working? (Você acha que são necessários exames regulares de RNI para saber se o anticoagulante está funcionando bem?)	0.9	0.95	0.95	
**Item 4** Is an INR value above your target range good for your general wellbeing? (Você acha que um valor de RNI acima da sua faixa ideal é bom para o seu bem-estar geral?)	0.85	0.9	0.95	
**Item 5** Is it possible for INR values below your target range to be bad for your health? (Você acha que resultados de RNI abaixo da sua faixa ideal são ruins para a sua saúde?)	0.9	0.95	1	
**Item 6**a Is it possible for what you eat to affect your warfarin therapy? (Você acha que o que você come afeta o seu tratamento com varfarina?)	0.85	0.95	0.95	
**Item 6**b If you answered ‘Yes’ above, list THREE foods that can affect your anticoagulant therapy. (Se você respondeu ‘Sim’ na questão anterior, você sabe dizer TRÊS alimentos que podem afetar o seu tratamento com anticoagulantes?)	0.9	1	0.9	
**Item 7** List one vitamin that can significantly affect your anticoagulant therapy. (Cite uma vitamina que pode afetar significativamente o seu tratamento com anticoagulante.)	0.85	0.9	0.9	

I-CVI=item content validity index; INR=international normalized ratio; RNI=razão normalizada internacional; S-CVI=scale content validity index

### Construct Validity

**Fig. 1:**
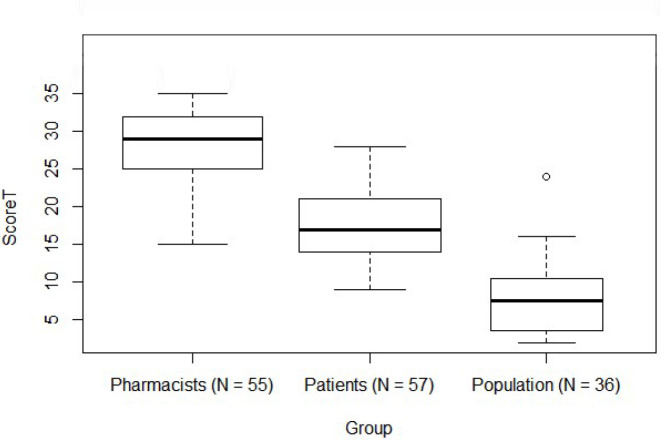
Total score comparison between groups/Tukey’s post hoc test showed significant differences (P<0.001) in comparing the means of all the groups (pharmacists vs. patients; pharmacists vs. population; patients vs. population).

There were significant differences between the scores of the three evaluated groups (N=148) in the construct validity through the analysis of contrasted groups (Figure 1). The total mean scores of the pharmacist group, patient group, and population group were 29 [25-32], 17 [14-21], and 7.5 [3.75-10.25], respectively (F=209.49, *P*<0.001). Tukey’s post-hoc test showed significant differences for all comparisons (*P*<0.001).

## DISCUSSION

We developed and validated the AKT-Br and obtained supporting evidence for validity and reliability. We showed the instrument is useful in a non-English setting to objectively assess patients’ knowledge on anticoagulation.

The relationship among patients’ knowledge and adherence to OAC has been discussed for more than 30 years^[9]^, with important impacts on patients’ clinical and economic outcomes^[12,28,29]^. Deshpande et al.^[30]^ showed a significant reduction in the total adjusted costs of treatment of $29,742 for adherent patients *vs*. $33.609 for non-adherent patients. Although the costs with medication were higher in adherent patients ($5,595 *vs*. $2,233), these were offset by the reduction in the medical costs ($23,544 *vs*. $30,485), which may include readmissions or hospitalisations. Fonseca et al.^[31]^ demonstrated that the average length of hospital stay of patients using dabigatran or warfarin is around 4.8 and 5.5 days, with costs of $9,803 and $9,755, respectively.

These figures highlight the key role of patients’ self-monitoring and the use of healthcare interventions/services. In this context, Obamiro et al.^[14]^ developed the AKT to provide an instrument capable of assessing a patient’s knowledge regarding her/his anticoagulant treatment, where, according to the score obtained by the patient, health professionals could carry out assertive and personalised educational interventions towards better outcomes. In addition, researchers can use the AKT to measure the potential benefits of different interventions in patients using OACs^[32]^.

The availability of this tool in other languages besides English — such as Italian, as previously validated by Magon et al.^[32]^, or in Brazilian Portuguese — also allowed us to standardise how the results are presented and to further compare data among populations of different countries. In Brazil, several tools that have been translated into Portuguese and validated are now available in clinical practice. Examples include the Morisky Medication Adherence Scale^[33]^ — an eight-item tool that assesses the therapeutic adherence of patients undergoing treatment for hypertension — and the Diabetes Quality of Life Measure^[34]^ — a 46-question instrument that evaluates the quality of life of type II diabetes patients.

An instrument’s validity is not limited to a measurement of its properties; it is also an interaction of the scale with the population being tested. The results, when represented by numbers, allow researchers to measure specific population phenomena^[35]^ that may vary according to the cultural-linguistic features of that population. In other words, an instrument is not always valid or applicable from one population to another, or from another language compared with the original. That is why the challenges related to tool validity in cross-cultural research are mainly due to content validity. A tool is valid when the content, criteria, and construct validity items are minimally met. Content validity reflects the degree of adequacy of the instrument that is being built in relation to the study population. This occurs through the discussion of expert panels (3-5 specialists) and qualitative approaches that measure the CVI^[19,20,24,36]^, as performed in our study.

Construct validity is assessed through hypothetical predictions usually supported by hypothesis tests using a group contrast approach^[23]^. Different results are expected in the evaluated groups, an outcome that allows researchers to confirm the capacity of the instrument to detect differences in the population^[20,36]^. This comparison among groups is not intended for clinical implications; it is only meant to assess validity. We validated the AKT-Br construct according to Terwee's recommendations^[37]^. Unidirectional ANOVA and Tukey’s post hoc analysis demonstrated a significantly higher level of knowledge in the pharmacist group compared with the patient group and in the patient group compared with the general population. These results are in accordance with the findings of Obamiro et al.^[14]^ and Magon et al.^[32]^, who, in addition to expecting a higher score from the pharmacist group (specialists), presented significant differences among all group comparisons. These data strengthen the theory of the group comparison method for construct validity^[23]^, where the different levels of knowledge about anticoagulation could be stratified, validating the contractor’s ability to distinguish them. Finally, the AKT-Br presented a positive and significant correlation coefficient in the test-retest analysis, reaffirming the stability and reliability of the instrument at different times of application. This implies that the tool can be useful to provide consistent scores over time in a stable group of patients. AKT-Br items are interrelated, measuring the same construct, which is similar to the results obtained in the original research^[14]^.

### Limitations

The results obtained with the AKT-Br tool highlight the validity of its translation and cross-cultural adaptation to Brazilian Portuguese. Nonetheless, our study has some limitations. First, it lacks a criterion validity using a measure of adherence (*e.g*., Morisky score) and testing *a priori* framework, where patients with higher adherence should be those with greater knowledge. The data from the group of patients was obtained from a tertiary hospital in Curitiba, Paraná (South Region of Brazil), who may not reflect the cultural features of the entire country nor be representative of the Brazilian population taking OAC. Further analyses can be performed in other regions to guarantee the validity of the tool. In addition, some educational limitations from the population led the interviewer to assist in the interpretation of the questions, a factor that may generate bias. In addition, 90% (n=50) of the evaluated patients were warfarin users. This may imply a possible selection bias, which is compatible with the convenience sampling or a need to a readdress the management strategy of Brazilian patients prescribed OAC. This factor may also limit the assessment of data from patients only using DOAC. The main objective of the study was to validate the AKT-Br as a psychometric analysis tool. However, clinicians can also benefit from the development of a “scale of knowledge” according to the obtained scores. For example, to minimise adherence issues and OAC adverse events, patients who score between five and 10 points (out of 35) probably need to receive different educational interventions compared to those who score 20-25 points. This is especially important in regions or countries with greater socioeconomic inequalities.

## CONCLUSION

We showed that the developed AKT-Br is a valid psychometric tool for Brazilian Portuguese. This tool may enhance the quality of life and care of patients using OAC by minimising adverse effects and improving adherence to treatment through tailored educational interventions. Thus, we strongly recommend its routine use in clinical practice. Assessing the knowledge of DOAC users should be better addressed in the future.

**Table:** 

Authors' roles & responsibilities	
FFM	Substantial contributions to the conception or design of the work; or the acquisition, analysis, or interpretation of data for the work; drafting the work or revising it critically for important intellectual content; agreement to be accountable for all aspects of the work in ensuring that questions related to the accuracy or integrity of any part of the work are appropriately investigated and resolved; final approval of the version to be published
VLF	Substantial contributions to the conception or design of the work; or the acquisition, analysis, or interpretation of data for the work; agreement to be accountable for all aspects of the work in ensuring that questions related to the accuracy or integrity of any part of the work are appropriately investigated and resolved; final approval of the version to be published
AMM	Substantial contributions to the conception or design of the work; or the acquisition, analysis, or interpretation of data for the work; drafting the work or revising it critically for important intellectual content; final approval of the version to be published
GLM	Substantial contributions to the conception or design of the work; or the acquisition, analysis, or interpretation of data for the work; agreement to be accountable for all aspects of the work in ensuring that questions related to the accuracy or integrity of any part of the work are appropriately investigated and resolved; final approval of the version to be published
CJC	Substantial contributions to the conception or design of the work; or the acquisition, analysis, or interpretation of data for the work; agreement to be accountable for all aspects of the work in ensuring that questions related to the accuracy or integrity of any part of the work are appropriately investigated and resolved; final approval of the version to be published
FST	Substantial contributions to the conception or design of the work; or the acquisition, analysis, or interpretation of data for the work; drafting the work or revising it critically for important intellectual content; agreement to be accountable for all aspects of the work in ensuring that questions related to the accuracy or integrity of any part of the work are appropriately investigated and resolved; final approval of the version to be published
RP	Substantial contributions to the conception or design of the work; or the acquisition, analysis, or interpretation of data for the work; agreement to be accountable for all aspects of the work in ensuring that questions related to the accuracy or integrity of any part of the work are appropriately investigated and resolved; final approval of the version to be published

## SUPPLEMENTARY MATERIAL

Appendix 1 - AKT-Br - Validation of the Brazilian version of Anticoagulation Knowledge Tool (AKT-Br - Validação da versão brasileira da ferramenta de conhecimento sobre anticoagulantes)

### Validação da ferramenta de conhecimento sobre anticoagulantes

#### Introdução

Agradecemos sua colaboração em preencher este questionário e por disponibilizar seu tempo para participar desta pesquisa. Sua participação irá ajudar a validar esta ferramenta, que será útil no cuidado de pessoas que usam medicamentos anticoagulantes orais. Fique tranquilo de que todas as suas respostas, incluindo informações pessoais, serão mantidas anônimas e de que o sigilo e a confidencialidade dos seus dados estarão garantidos.

Instruções para preenchimento do questionário se você está atualmente tomando um medicamento anticoagulante oral:

- Por favor, preencha as questões a seguir de modo que expressem o mais exatamente possível suas opiniões e da melhor maneira os seus conhecimentos.
- Se você não souber a resposta de uma questão, não há problemas, por favor, escreva ‘Eu não sei’ no espaço fornecido.
- Se você não estiver certo da resposta de uma pergunta de múltipla escolha, por favor, marque a opção ‘Não tenho certeza’.

##### Informações demográficas

1. Qual o seu gênero?
  - Masculino
  - Feminino
2. Quantos anos você tem? _____________ anos
3. Qual é o seu nível de escolaridade?
  - Ensino fundamental
  - Ensino médio
  - Ensino técnico
  - Ensino superior
  - Pós-graduação
  - Sem formação educacional
4. Há quanto tempo você está tomando anticoagulante?
  - Menos de 3 meses
  - 3-12 meses
  - 1-2 anos
  - Mais de 2 anos
  - Eu não estou tomando anticoagulante

##### Seção A: Conhecimento sobre anticoagulação

###### 2.1. Questões gerais

1. Qual é o nome do seu medicamento anticoagulante?
  ____________________________________________________________
2. Por que seu médico prescreveu esse medicamento para você?
  ____________________________________________________________
3. Você sabe como esse medicamento age em seu corpo?
  ____________________________________________________________
4. Quantas vezes ao dia você precisa tomar esse medicamento?
  ____________________________________________________________
5. Por quanto tempo você precisa tomar esse medicamento (por exemplo, 3 meses, 6 meses, por toda a vida)?
  ____________________________________________________________
6. Por que é importante tomar esse medicamento exatamente como o seu médico lhe explicou?
  ____________________________________________________________
7. É importante tomar esse medicamento no mesmo horário todos os dias? a) Sim b) Não c) Não tenho certeza
8. Você acha que existirá algum problema se você dobrar a dose do anticoagulante caso tenha esquecido de tomar a dose anterior? a) Sim b) Não c) Não tenho certeza
9. Você acha que esquecer uma dose do anticoagulante pode piorar a sua doença? a) Sim b) Não c) Não tenho certeza
10. Você acha apropriado parar de tomar o anticoagulante quando você se sente melhor? a) Sim b) Não c) Não tenho certeza
11. Você acha seguro tomar medicamentos anti-inflamatórios, como ibuprofeno (Alivium^®^, Ibupril^®^ ou Advil^®^), enquanto você está tomando esse anticoagulante? a) Sim b) Não c) Não tenho certeza
12. Você acha seguro tomar suplementos vitamínicos ou ervas medicinais com esse medicamento sem consultar seu médico? a) Sim b) Não c) Não tenho certeza
13. Você acha que existe algum benefício em tomar doses acima da recomendada pelo seu médico? a) Sim b) Não c) Não tenho certeza
14. Você acha que tomar bebidas alcoólicas em grandes quantidades com esse medicamento aumenta o risco de efeitos colaterais? a) Sim b) Não c) Não tenho certeza
15. Você informaria um cirurgião, dentista ou outro profissional de saúde de que está tomando esse medicamento antes de realizar uma cirurgia ou um procedimento? a) Sim b) Não c) Não tenho certeza
16. É importante que todos os profissionais de saúde pelos quais você é acompanhado saibam que você faz uso de anticoagulante? a) Sim b) Não c) Não tenho certeza
17. Qual é o efeito colateral mais importante que seu anticoagulante pode causar?
  ____________________________________________________________
18. Cite TRÊS sinais de efeitos colaterais para os quais você deve estar atento quando usa anticoagulante:
  ____________________________________________________________
19. Cite TRÊS coisas que você pode fazer para reduzir os riscos de efeitos colaterais dos anticoagulantes:
  ____________________________________________________________
20. Se acidentalmente você tomasse uma dose de anticoagulante muito acima da prescrita, o que você faria
  ____________________________________________________________

###### 2.2 Seção B

1. Qual é o valor de RNI ideal para você?
2. Qual foi seu último resultado de RNI?
3. Você acha que são necessários exames regulares de RNI para saber se o anticoagulante está funcionando bem? a) Sim b) Não c) Não tenho certeza
4. Você acha que um valor de RNI acima da sua faixa ideal é bom para o seu bem-estar geral? a) Sim b) Não c) Não tenho certeza
5. Você acha que resultados de RNI abaixo da sua faixa ideal são ruins para a sua saúde? a) Sim b) Não c) Não tenho certeza
6a. Você acha que o que você come afeta o seu tratamento com varfarina? a) Sim b) Não c) Não tenho certeza
6b. Se você respondeu ‘Sim’ na questão anterior, você sabe dizer TRÊS alimentos que podem afetar o seu tratamento com anticoagulantes?
  ____________________________________________________________
7. Cite uma vitamina que pode afetar significativamente o seu tratamento com anticoagulante.
  ____________________________________________________________

### TERMO DE CONSENTIMENTO LIVRE E ESCLARECIDO

Nós, Prof. Dr. Roberto Pontarolo, Dr. Vinicius Lins Ferreira, Prof. Dr. Gustavo Lenci Marques e Ms. Felipe Fernando Mainka, da Universidade Federal do Paraná, estamos convidando você, usuário de anticoagulante oral, a participar de um estudo intitulado “Tradução e adaptação transcultural para o português do Brasil e validação do questionário de avaliação do conhecimento de anticoagulantes orais (Oral Anticoagulation Knowledge Tool - AKT)”. A validação do questionário que avalia o conhecimento do paciente sobre seu anticoagulante oral poderá dar acesso a uma ferramenta de intervenção, proporcionando assim uma maior adesão ao tratamento e uma redução dos efeitos adversos causados pelo uso incorreto de anticoagulantes orais.

Os objetivos desta pesquisa são realizar a tradução e adaptação transcultural da ferramenta AKT para o português do Brasil e validá-la para avaliação do conhecimento da população brasileira em uso de anticoagulantes orais.

Caso você participe da pesquisa, será necessário que você preencha completamente o questionário, que consta de 32 questões e requer aproximadamente 20 a 30 min para conclusão; em caso de dúvidas ou desconforto em relação a alguma questão, você poderá solicitar auxílio do pesquisador.

Para tanto, o questionário será aplicado oportunamente no dia em que estiverem agendadas as consultas no ambulatório de anticoagulantes. O ambulatório fica localizado no Complexo Hospital de Clínicas da Universidade Federal do Paraná, Rua General Carneiro, 181, CEP: 80060-900, Curitiba/PR. Para responder ao questionário são necessários 20 a 30 min.

É possível que você experimente algum desconforto, principalmente relacionado a cansaço, durante o preenchimento do questionário ou até mesmo referente a alguma questão que possa lhe causar constrangimento; nesse caso, o pesquisador coloca-se à disposição para esclarecimentos.

Alguns riscos relacionados ao estudo podem ser referentes ao constrangimento durante a entrevista, oriundos de alguma questão que possa causar-lhe desconforto; nesse caso, o pesquisador coloca-se à disposição para esclarecimentos.

O benefício esperado com esta pesquisa é a validação de um questionário que a avalia o conhecimento do paciente sobre seu anticoagulante oral, podendo dar acesso a uma nova ferramenta de intervenção, o que pode proporcionar maior adesão ao tratamento e uma redução dos efeitos adversos causados pelo uso incorreto de anticoagulantes orais, embora nem sempre você seja diretamente beneficiado(a) por sua participação neste estudo.

Os pesquisadores Roberto Pontarolo, Vinicius Lins Ferreira, Gustavo Lenci Marques e Felipe Fernando Mainka, responsáveis por este estudo, poderão ser localizados para esclarecer eventuais dúvidas que você possa ter e fornecer-lhe as informações que queira, antes, durante ou depois do estudo, na Universidade Federal do Paraná - Campus III, Av. Pref. Lothário Meissner, 632, Jardim Botânico, CEP: 80210-170, Curitiba/PR; pelos e-mails pontarolo@ufpr.br, vinicius_lins1991@hotmail.com, gustavolencimarques@gmail.com e felipemainka@hotmail.com; e pelo telefone comercial (41) 3360-1894, no horário das 09:00 às 17:00 horas, de segunda à sexta-feira. Em situações de urgência e emergência relacionadas à pesquisa, os mesmos poderão ser contatados pelos telefones (41) 99181-6622 e (41) 99728-0962, disponíveis nas 24 horas, com acesso direto aos pesquisadores envolvidos.

Se você tiver dúvidas sobre seus direitos como participante de pesquisa, poderá contatar o Comitê de Ética em Pesquisa em Seres Humanos do Complexo Hospital de Clínicas da Universidade Federal do Paraná (CEP/HC/UFPR) pelo telefone (41) 3360-1041, das 08:00 às 14:00 horas, de segunda à sexta-feira. O CEP/HC/UFPR é formado por um grupo de indivíduos com conhecimentos científicos e não científicos que realizam a revisão ética inicial e continuada do estudo de pesquisa para mantê-lo seguro e proteger seus direitos.

A sua participação neste estudo é voluntária e se você não quiser mais fazer parte da pesquisa, poderá desistir a qualquer momento e solicitar que lhe devolvam este Termo de Consentimento Livre e Esclarecido assinado.

As informações relacionadas ao estudo poderão ser conhecidas por pessoas autorizadas. No entanto, se qualquer informação for divulgada em relatório ou publicação, isto será feito sob forma codificada, para que a sua identidade seja preservada e seja mantida sua confidencialidade.

O material obtido (os questionários) será utilizado unicamente para esta pesquisa e será destruído/descartado ao término do estudo, dentro de 5 (cinco) anos.

As despesas necessárias para a realização da pesquisa não são de sua responsabilidade e você não receberá qualquer valor em dinheiro pela sua participação.

Você terá garantia de que desconfortos relacionados ao processo de preenchimento do questionário serão tratados no local.

Quando os resultados forem publicados, seu nome não aparecerá, e sim um código.

Rubricas:

Participante da pesquisa e/ou responsável legal ____________

Pesquisador responsável ou quem aplicou o TCLE ____________

Eu,________________________________________, li este Termo de Consentimento e compreendi a natureza e o objetivo do estudo do qual concordei em participar. A explicação que recebi menciona os riscos e benefícios. Eu entendi que sou livre para interromper minha participação a qualquer momento sem justificar minha decisão e sem qualquer prejuízo para mim nem para meu tratamento ou atendimento ordinários que eu possa receber de forma rotineira na Instituição. Eu entendi o que não posso fazer durante a pesquisa e fui informado que serei atendido sem custos para mim se eu apresentar algum problema diretamente relacionado ao desenvolvimento da pesquisa.

Eu concordo voluntariamente em participar deste estudo.

____________________________________________________________

Nome por extenso e legível do Participante e/ou Responsável Legal

____________________________________________________________

Assinatura do Participante e/ou Responsável Legal

Declaro que obtive, de forma apropriada e voluntária, o Consentimento Livre e Esclarecido deste participante e/ou seu representante legal para a participação neste estudo.

____________________________________________________________

Nome extenso do Pesquisador e/ou quem aplicou o TCLE

____________________________________________________________

Assinatura do Pesquisador e/ou quem aplicou o TCLE

Curitiba, ______________________________________

Rubricas:

Participante da pesquisa e/ou responsável legal ____________

Pesquisador responsável ou quem aplicou o TCLE ____________
